# Measurement of Cephalic Indices in Older Children and Adolescents of a Nigerian Population

**DOI:** 10.1155/2014/527473

**Published:** 2014-04-08

**Authors:** Babatunde Olayemi Akinbami

**Affiliations:** Departments of Oral and Maxillofacial Surgery and Human Anatomy, University of Port Harcourt, Port Harcourt 500004, Rivers State, Nigeria

## Abstract

*Background*. A study on the cephalic index was carried out on subjects from school students in Ogbia tribe of Bayelsa state in the Niger Delta region of Nigeria. This study determined the cephalic indices among the school students. In the present study, seven hundred subjects were randomly selected from secondary schools comprising 350 males and 350 females, respectively, with age range from 11–20 years, with both parents and grandparents of Ogbia tribe. The length and breadth of the head were measured using a spreading caliper from standard bony landmarks. The measurable point for head length was measured between the glabella and inions while the head breadth was the widest biparietal diameter. The cephalic index was head breadth divided by the head length and multiplied by 100. The result showed that the mean cephalic index was 76.56. Males had a cephalic index of 77.21, while females had a cephalic index of 76.50. Based on this study, 78.68% of individuals were mesocephalic, 11.4% dolichocephalic, 9.0% Brachycephalic, and 0.43% hyperbrachycephalic. This research showed that the school students have mesocephalic phenotype. The data from this research will be useful in anthropology, genetics, forensic medicine, and clinical practice.

## 1. Introduction

Cephalic index is a useful anthropometric parameter utilized in the determination of racial variations [1]. It is also used to determine sexual differences especially in individuals whose identities are unknown [2]. It is one of the clinical anthropometric parameters recognized in the investigation of craniofacial skeletal deformities and brain development because of its validity and practicality [3].

Cephalic index is the most frequently investigated craniofacial parameter as it utilizes the length and breadth of the head which are useful indices in the study of secular trend [4, 5]. The ratio of the maximum head breadth to the maximum head length can be used to measure the size of the head [6]; cephalic index gives an idea of how genetic characters are transmitted between parents, offspring, and siblings [1]. It is inherited in a unitary fashion [7]. Isolated or syndromic craniosynostosis, primary microcephaly, and hydrocephalus are pathological disorders which manifest with abnormal cephalic indices in addition to other features.

On basis of cephalic index, head shapes are grouped into four international categories, which are dolichocephal, brachycephal, mesocephal, and hyperbrachycephal [2, 8]. The research aimed at comparing the cephalic index between the two genders in a selected population and at determining a baseline value of cephalic index which could be vital in forensic, anthropological, and clinical studies.

## 2. Materials and Methods

**Figure 1:**
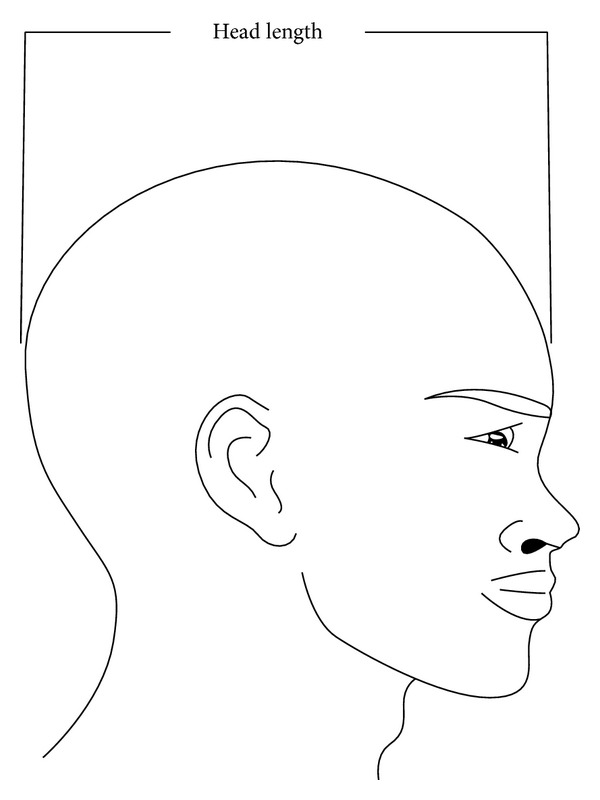
Diagram showing the measurement of head length.

**Figure 2:**
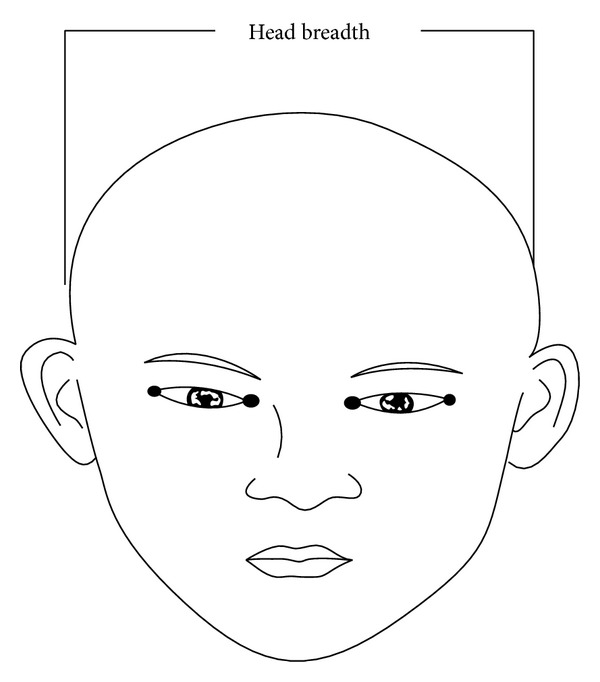
Diagram showing the measurement of head breadth.

The methodology was designed to determine the relation that exists between the cephalic index of 700 subjects from a selected population (350 males and 350 females). Head breadth and head length are measured with a standardized spreading (vernier) caliper. Values obtained for these variables were documented. The subjects were selected randomly, one out of every eight students in each level of study, from seven schools in Bayelsa state, Nigeria. 100 students (50 males and 50 females) were selected from each school. Subjects selected for the study were between the age range of 11–20 years. Subjects with craniofacial trauma were excluded from this study. All measurements were taken with the subjects sitting on a chair in a relaxed mood and the head in an anatomical position. The head length was measured by placing one end at the glabella and the other end at the inions [9] (Figure 1). The two points were then measured using a meter rule to the nearest centimeter (cm), with an accuracy of 0.10. The head breadth was measured as the maximum transverse diameter between the two fixed points over the parietal bones [10] by the use of a spreading caliper (Figure 2). The two fixed points were then measured using a meter rule to the nearest centimeter (cm), with an accuracy of 0.10. The cephalic index was calculated for each using the following equation [11]. Biparietal diameter divided by length of cranium and multiplied by 100. The slide of the caliper was properly tightened; this is to avoid a shift in the jaws of the caliper thereby bringing about errors due to parallax. To avoid a large degree of inaccuracy, the readings were measured with an accuracy of 0.10 cm. All measurements were done by the author and repeated twice on each patient to ensure validation.

## 3. Statistical Analysis

Data collected from the measurement of the cephalic indices for both male and female subjects were subjected to statistical analysis to show the measurement of the central tendency for the cephalic indices. The data was analyzed with SPSS version 16 to calculate the mean, standard deviation (SD), and standard error (SE). *Z*-test was used for comparison between genders.

## 4. Result

The mean maximum head breadth in male and female school students was 14.23 cm and 13.67 cm, respectively (Table 4), while the mean maximum head lengths for males and females were found to be 18.39 cm and 17.97 cm, respectively (Table 5). The mean cephalic indices in males and females were 77.21 and 76.50, respectively (Table 6).

The school students irrespective of sex had mean maximum head breadth, mean maximum head length, and mean cephalic index which were 14.05 cm, 18.18 cm, and 76.86, respectively (Table 7).

From Table 1, the majority of males and females had maximum head breadth that fell within the range from 14.00 to 14.99 and 13.00 to 13.99, respectively. The lowest percentage for both genders fell within the range from 11.00 to 11.99.

The highest percentage of maximum head breadth for both males and females was 81.14% and 48.86, respectively.

From Table 2, the majority of both males and females had maximum head length that fell within the range from 18.00 to 18.99, while the lowest percentage fell within the range from 20.00 to 20.99.

The highest percentage of maximum head length for both male and female was 74.86%.

From Table 3, the majority of both males and females had cephalic index that fell within the range from 75.00 to 79.99, while both males and females lowest population fell within the ranges from 60.00 to 69.99, 85.00 to 89.99, and 60.00 to 64.49, respectively.

The highest percentage of cephalic index for both males and females was 80.86% and 76.29%, respectively.

From Table 4, the mean values of maximum head breadth for both males and females were 14.32 cm and 13.67 cm, respectively. This shows a significant difference between the males and females because the confidence interval (CI) does not fall within the same range. Therefore, 14.26–14.38 cm and 11.72–15.26 were acceptable as normal head breadth for males and females in this selected population.

From Table 5, the mean values of maximum head length for both males and females were 18.77 cm and 17.97 cm, respectively. The result shows that the difference is significant because confidence interval does not fall within the same range. Set values of 16.82–18.92 cm and 17.21–18.73 cm were acceptable as normal head length for males and females.

From Table 6, mean values of cephalic index for both males and females were 77.21 and 76.50, respectively. There was significant difference between the male and female cephalic index. Set values of 74.80–79.62 and 78.83–79.17 were acceptable as normal cephalic for males and females.

From Table 7, the *Z*-calculated is greater than *Z*-tabulated (*Z*-cal > *Z*-tab); thus null hypothesis is rejected. This implies that the result between the male and female shows significant difference.

From Table 8, for all the subjects, the mean values for the maximum head breadth, maximum head length, and cephalic indices were 14.05 cm, 18.18 cm, and 76.86, respectively. On a final analysis, based on the baseline values, 78.6% of subjects were mesocephalic, 11.4% dolichocephalic, 9% brachycephalic, and 0.4% hyperbrachycephalic.

## 5. Discussion

Patency of the metopic and sagittal as well as coronal and lambdoid sutures is responsible for the adequate growth of cranial vault/base in the anterior-posterior and transverse directions, respectively, based on Virchow's law of parallel and perpendicular bone expansion. Metopic suture fuses between 3 and 9 months, while the others fuse between 22 and 39 months [12]. Premature fusion of the sagittal suture will increase the entire length of the head anterior-posterior while only the length of the anterior is reduced in early metopic fusion. Early fusion of bilateral coronal sutures results in increased biparietal diameter and reduced head length (classical brachycephaly) [1]. This is worsened when the lambdoid sutures are affected. The growth of the face is dependent on the base of the skull and it is also affected when these sutures fuse early [12].

This study showed that the cephalic index of males was significantly higher than those of females (*P* = 0.04); the reason for this difference cannot be immediately explained but it agrees with sexual dimorphism as reported by Olotu et al. [13]. The cephalic index of this study was lower than Fawehinmi's study in Port Harcourt, Nigeria, with 79.80, Fawehinmi et al. [14].

Oladipo and Olotu determined the cephalic index for Ijaw male and female as 80.98 and 78.24, respectively. They also worked on the cephalic index of Igbo male and female as 79.09 and 76.83, respectively. Their findings were higher than that of the present study. The reason for this difference cannot be immediately explained but hypothetically, it may be due to environmental, genetic, or even nutritional cause. Factors involved in suture patency and normal bone growth including hormonal factors, maternal smoking, and hyperthyroidism have been linked to early fusion of sutures. Alterations in the level of fibroblastic growth factor type 3 receptor genes and transcriptase factor receptor gene have also been attributed.

The studies carried out by Oladipo and Olotu on the cephalic indices of Ijaw male and female indicated that they fall into the brachycephalic group and mesocephalic group, respectively, while the Igbo males and females fall in the mesocephalic group [10].

Cephalic indices from this present study were lower than the cephalic indices documented by Shah and Jadhav [1] study in India and del Sol [8] in Chile. Cephalic indices from this present study were also lower than a study in native Fars males with 84.8 [11].

In the present study, it was observed that there was gender difference with males having a higher cephalic index compared to females. The reasons for this difference are not clear but may be attributed to the effect and interplay amongst growth, thyroid, and sex hormones. Investigation carried out on the cephalic indices of males and females of Gurung community in Nepal revealed a significant gender difference [15], with males having a cephalic index of 83.1 which is lower than females with cephalic index of 84.6. This implies that cephalic index can be higher in any sex depending on the peculiarity of the population under study.

In the present study, the dominant type of head shape was mesocephalic (78.6%). This finding is similar to other studies in del Sol, Chile, Bhargav and Kher study in central India [16], Oladipo's study of Igbo tribe of Nigeria [10], and Fawehinmi's study in Port Harcourt, Nigeria [14]. Dominant type of head from this study is not similar to a study in India [17] in which 58.5% of the population was dolichocephalic.

It is worthy to note that the percentage of head type also varies in different population. In a study on 50 individuals in the IX Region of Chile, del Sol reported that 77% of the individuals were mesocephalic, 28% brachycephalic, 4% hyperbrachycephalic, and 2% dolichocephalic.

Besides, in a study in India, it was shown that 41% of the students were mesocephalic, 37% brachycephalic, 14% hyperbrachycephalic, and 7% dolichocephalic (Shal and Jadhav). In the present study 78.6% of subjects were mesocephalic, 11.4% dolichocephalic, and 9% brachycephalic, while 0.4% of subjects were hyperbrachycephalic.

The percentage of head shape observed in the different study may be connected with heredity factor. Environment may also have a secondary effect [11]. The kind of diet could also play a role in influencing the dominant head shape. Head shapes can also change from one generation to the other.

Brachycephalization is thought to be due to relative higher increase in the head breadth in comparison with the head length as a result of improvement in nutrition [4]. Variation in cephalic indices between and within population has been attributed to a complex interaction between genetic and environmental factors [18].

Thus the males and females in this study belong to the same tribe in Nigeria and they have same origin but there is a significant difference between their cephalic indices. However, both genders were said to be mesocephalic. The results of this study are expected to be of importance to anthropologist and forensic scientist and in clinical practice as in cephalopelvic disproportion (CPD) and craniosynostosis. It may also serve as the basis of comparison for further studies on other Nigerian ethnic groups or minority tribes. Further studies on gene variation are required to ascertain the specific genetic factors responsible for differences in cephalic indices among sexes, tribes, and races. Levels of intelligence quotient and degree of mental retardation will also need to be assessed to determine the effect of abnormal alterations in cephalic indices as well as the relationship with brain growth.

In conclusion, this study shows a significant increase of mesocephalic and dolichocephalic and a significant decrease of brachycephalic and hyperbrachycephalic head shape in both sexes. This result suggests a continuity of debrachycephalization process.

## Figures and Tables

**Table 1:** Gender distribution of values of maximum head breadth (MHB) in the selected population.

Range/cm	Frequency *N* (%)			
	Male	%	Female	%
11.00–11.99	2	0.57	1	0.29
12.00–12.99	8	2.29	42	12.00
13.00–13.99	48	13.71	171	48.86
14.00–14.99	284	81.14	130	37.04
15.00–15.99	8	2.29	6	1.71

Total	350	100	350	100

**Table 2:** Gender distribution of values of maximum head length (MHL).

Range/cm	Frequency *N* (%)			
	Male	%	Female	%
16.00–16.99	10	2.86	29	8.29
17.00–17.99	50	14.26	147	42.00
18.00–18.99	262	74.86	157	44.86
19.00–19.99	25	7.14	16	4.57
20.00–20.99	3	0.86	1	0.29

Total	350	100	350	100

**Table 3:** Gender distribution of values of the cephalic indices (CI).

Range/cm	Frequency *N* (%)			
	Male	%	Female	%
60.00–60.49	0	0	1	0.29
65.00–69.99	1	0.29	2	0.57
70.00–74.49	25	7.14	55	15.71
75.00–79.99	283	80.86	267	76.29
80.00–84.99	40	11.43	23	6.57
85.00–89.99	1	0.29	2	0.57

Total	350	100	350	100

**Table 4:** Mean, standard deviation (SD), and standard error (SE) of maximum head breadth.

Parameters	Males	Females
Mean (*x*) in cm	14.32	13.67
Standard deviation	0.6	1.95
Standard error (SE)	0.03	0.1
Sample size (*N*)	350	350

*P* < 0.05 (0.002).

**Table 5:** Mean, standard deviation (SD), and standard error (SE) of maximum head length (MHL).

Parameters	Males	Females
Mean (cm)	18.37	17.97
Standard deviation	0.55	0.76
Standard error (SE)	0.03	0.04
Sample size (*N*)	350	350

*P* < 0.05 (0.001).

**Table 6:** Mean, standard deviation (SD), and standard error (SE) of cephalic index (CI).

Parameters	Males	Females
Mean (%)	77.21	76.50
Standard deviation (SD)	2.41	2.67
Standard error (SE)	0.13	0.14
Sample size (*N*)	350	350

*P* < 0.05 (0.04).

**Table 7:** *Z*-test result for the maximum head breadth (MHB), maximum head length (MHL), and cephalic indices (CI).

Comparison	*Z*-calculated	*Z*-tabulated	Evaluated level
MHB	7.22	1.96	0.05
MHL	6.67	1.96	0.05
CI	5.92	1.96	0.05

**Table 8:** Overall mean and standard deviation (SD) of maximum head breadth (MHB), maximum head length (MHL), and cephalic indices (CI) of the selected population.

Parameters	MHB (cm)	MHL (cm)	CI
Mean	14.05	18.18	76.86
Standard deviation	0.71	0.62	2.41
Sample size	700	700	700
